# Metagenomics-Toolkit: the flexible and efficient cloud-based metagenomics workflow featuring machine learning-enabled resource allocation

**DOI:** 10.1093/nargab/lqaf093

**Published:** 2025-07-17

**Authors:** Peter Belmann, Benedikt Osterholz, Nils Kleinbölting, Alfred Pühler, Andreas Schlüter, Alexander Sczyrba

**Affiliations:** IBG-5: Computational Metagenomics, Institute of Bio- and Geosciences (IBG), Research Center Jülich GmbH, D-52428 Jülich, Germany; Computational Metagenomics Group, Faculty of Technology and Center for Biotechnology (CeBiTec), Bielefeld University, Universitätsstrasse 25, D-33615 Bielefeld, Germany; IBG-5: Computational Metagenomics, Institute of Bio- and Geosciences (IBG), Research Center Jülich GmbH, D-52428 Jülich, Germany; Computational Metagenomics Group, Faculty of Technology and Center for Biotechnology (CeBiTec), Bielefeld University, Universitätsstrasse 25, D-33615 Bielefeld, Germany; IBG-5: Computational Metagenomics, Institute of Bio- and Geosciences (IBG), Research Center Jülich GmbH, D-52428 Jülich, Germany; Genome Research of Industrial Microorganisms, Center for Biotechnology (CeBiTec), Universitätsstrasse 27, D-33615 Bielefeld, Germany; Computational Metagenomics Group, Faculty of Technology and Center for Biotechnology (CeBiTec), Bielefeld University, Universitätsstrasse 25, D-33615 Bielefeld, Germany; IBG-5: Computational Metagenomics, Institute of Bio- and Geosciences (IBG), Research Center Jülich GmbH, D-52428 Jülich, Germany; Computational Metagenomics Group, Faculty of Technology and Center for Biotechnology (CeBiTec), Bielefeld University, Universitätsstrasse 25, D-33615 Bielefeld, Germany

## Abstract

The metagenome analysis of complex environments with thousands of datasets, such as those in the Sequence Read Archive, requires substantial computational resources for it to be completed within a reasonable time frame. Efficient use of infrastructure is essential, and analyses must be fully reproducible with publicly available workflows to ensure transparency. Here, we introduce the Metagenomics-Toolkit, a scalable, data-agnostic workflow that automates the analysis of short and long metagenomic reads from Illumina and Oxford Nanopore Technology devices, respectively. The Metagenomics-Toolkit provides standard features such as quality control, assembly, binning, and annotation, along with unique capabilities including plasmid identification, recovery of unassembled microbial community members, and discovery of microbial interdependencies through dereplication, co-occurrence, and genome-scale metabolic modeling. Additionally, the Metagenomics-Toolkit includes a machine learning-optimized assembly step that adjusts peak RAM usage to match actual requirements, reducing the need for high-memory hardware. It can be executed on user workstations and includes optimizations for efficient cloud-based cluster execution. We compare the Metagenomics-Toolkit with five widely used metagenomics workflows and demonstrate its capabilities on 757 sewage metagenome datasets to investigate a possible sewage core microbiome. The Metagenomics-Toolkit is open source and available at https://github.com/metagenomics/metagenomics-tk.

## Introduction

Metagenomics addresses genome analyses of microbiome members residing in targeted environments and habitats. What complicates matters is that many microorganisms, especially in complex microbiomes, are currently unknown and often have not yet been cultivated. However, they may fulfill important functions in their respective ecosystem. These so far non-cultivable organisms are often referred to as the “microbial dark matter” [1]. Since the microbial dark matter represents a large fraction of microbiomes in almost all environments [2], large-scale metagenomic analyses of thousands of samples have been carried out in environments such as the ocean [3], soil [4], and humans [5], to further explore these unknown organisms by generating metagenome assembled genomes (MAGs). It is to be expected that more large-scale studies will be conducted in the future due to the increasing amount of sequencing data [6].

Already metagenomic analysis of single samples generates a large amount of data and inherently needs substantial compute resources, especially for processing steps such as metagenome assembly or annotation. While single samples can still be managed on individual workstations, the processing of metagenomic data from complex environments, such as anaerobic digestion or sewage microbiomes, often involving hundreds or thousands of samples, requires a lot more resources. These can usually only be provided in the form of high performance clusters or cloud computing services. Cloud computing has rapidly become an indispensable resource for researchers and organizations who have to store, manage, and analyze large amounts of data. The primary benefits of cloud computing include the ability to adapt resources through dynamic adjustment, which involves increasing or decreasing the number of virtual machines (VMs), so-called cloud compute instances based on the volume of data or computational demands. This system also enhances analysis speed by distributing workloads across multiple instances. Additionally, it offers the capability to customize instances, thereby ensuring the suitability of these instances for various tasks within a workflow. These characteristics make cloud-based solutions both flexible and cost-effective, as they allow for efficient handling of variable computational demands. However, to use resources on cloud systems, appropriate computational workflows have to manage and scale on a plethora of cloud compute instances. The available resources should be used in an efficient way by specifying the required resources as close as possible to what is actually needed in order to reduce the costs in public clouds and facilitate the execution of multiple tools in parallel. In general, it can be said that processing large amounts of metagenomic data manually can be time-consuming and prone to errors. Consequently, it is unreasonable to process multiple metagenomes simultaneously without some sort of automated execution of individual computational processing steps as, for example, provided by workflow engines.

The analysis or re-analysis of metagenomic samples, especially on a large scale, is not only computationally challenging, but also places an emphasis on the explorability of the data. Analysis results from hundreds or thousands of samples should be easy to explore and comprehend, especially for users without a background in computer science. For this user group, computed results should not be solely available in the form of text files. The explorability becomes even more important in the case of comparative analyses. Comparative analyses of predicted coding sequences, their annotation, biological processes, or abundances of MAGs will become slow and tedious without a sufficiently fast database engine and suitable visualization.

To enable enhanced reproducibility and scaling capabilities, we developed a workflow using the Nextflow [7] workflow engine which tackles the aforementioned challenges, allows the application on single workstations, and optimizes the application in cloud environments. We refer to this workflow as the “Metagenomics-Toolkit”. The Metagenomics-Toolkit offers novel analysis capabilities compared with other workflows in the form of sample-wise consensus-based plasmid detection and fragment recruitment, as well as cross-dataset dereplication and co-occurrence analysis enhanced by metabolic modeling. To broaden accessibility and usability for users without a computer science background, the Metagenomics-Toolkit is also available through a user-friendly web-based interface, powered by the Cloud-based Workflow Manager (CloWM) [8] service. Toolkit outputs can be investigated via the Exploratory MetaGenome Browser (EMGB) [9] web application to collate, integrate, and visualize Metagenomics-Toolkit results in a user-friendly, graphical format.

In addition, we applied a machine learning approach to predict RAM requirements of an assembler based on the characteristics of the input dataset. This allows more precise resource allocation, which may result in a reduction of the requested RAM and, in certain instances, the elimination of the necessity for dedicated high-memory hardware. Furthermore, this method could be adapted to other bioinformatics tools in the future to optimize their resource consumption.

Existing metagenomic workflows have their strengths and weaknesses, often focusing on a specific metagenomic analysis, offering support for multiple input types, such as long or short reads, or optimizing for specific computing environments. For example, MetaGEM [10] and MetaWrap [11] focus on a specific metagenomic topic, such as genome-scale metabolic modeling or a superior bin extraction algorithm. The MUFFIN [11, 12] workflow allows the incorporation of transcriptomic data, and nf-core/MAG [13] allows the combination of short and long reads and the incorporation of grouping information to perform co-assembly and binning. SequeezeMeta [14] is a software that can run on desktop computers with low resources. However, these workflows are either not designed for cross-dataset analyses, such as dereplication on thousands of samples, or they are not optimized for cloud-based cluster systems, which can limit their scalability. We compared the mentioned workflows in terms of their implemented features in four categories: “Sequence Data Input”, “Analysis Options”, “Data Handling”, and “Other Features”, highlighting novel features but also reporting features that will be implemented in the future.

To demonstrate the various analysis capabilities of the Metagenomics-Toolkit, we reanalyzed metagenomic datasets of untreated sewage, mainly collected by the Global Sewage Surveillance project. The examination of metagenome datasets from untreated sewage samples allows, for example, monitoring of the distribution of genes of interest, such as antimicrobial resistance genes [15]. Here we focus on the detection of the sewage core microbiome, i.e. species with a global distribution, and, most importantly, the full reproducibility and automation of our analysis, which will be useful for continuous tasks such as the monitoring of antibiotic resistance genes or pathogenic organisms on a global scale. All results generated by our workflow are publicly available for further investigation (see “Data availability”).

## Materials and methods

The Metagenomics-Toolkit developed in this study is based upon the Nextflow workflow engine, designed to streamline and automate the large-scale processing of metagenomic datasets. For the purposes of our analysis, we deployed Nextflow on a cluster within the de.NBI Cloud [16] infrastructure using BiBiGrid [17], an open source cluster management tool. This setup allowed us to efficiently handle the complex computational demands of the sewage core microbiome analysis. In this section, we provide a detailed account of the default configuration that was used and is provided as a best practice configuration of tools to the reader. In addition, we describe the machine learning strategy that was implemented to predict the RAM requirements of the assembler used.

### Utilizing nextflow and BiBiGrid on the de.NBI cloud infrastructure

We use an existing open-source workflow engine to efficiently process large amounts of metagenomic data. This domain-specific workflow engine Nextflow, developed for data-intensive bioinformatics, allows tools and subworkflows to be separated into modules. Nextflow workflows on clusters can read inputs and write results to compatible Amazon S3 API object storage systems.

**Figure 1.**
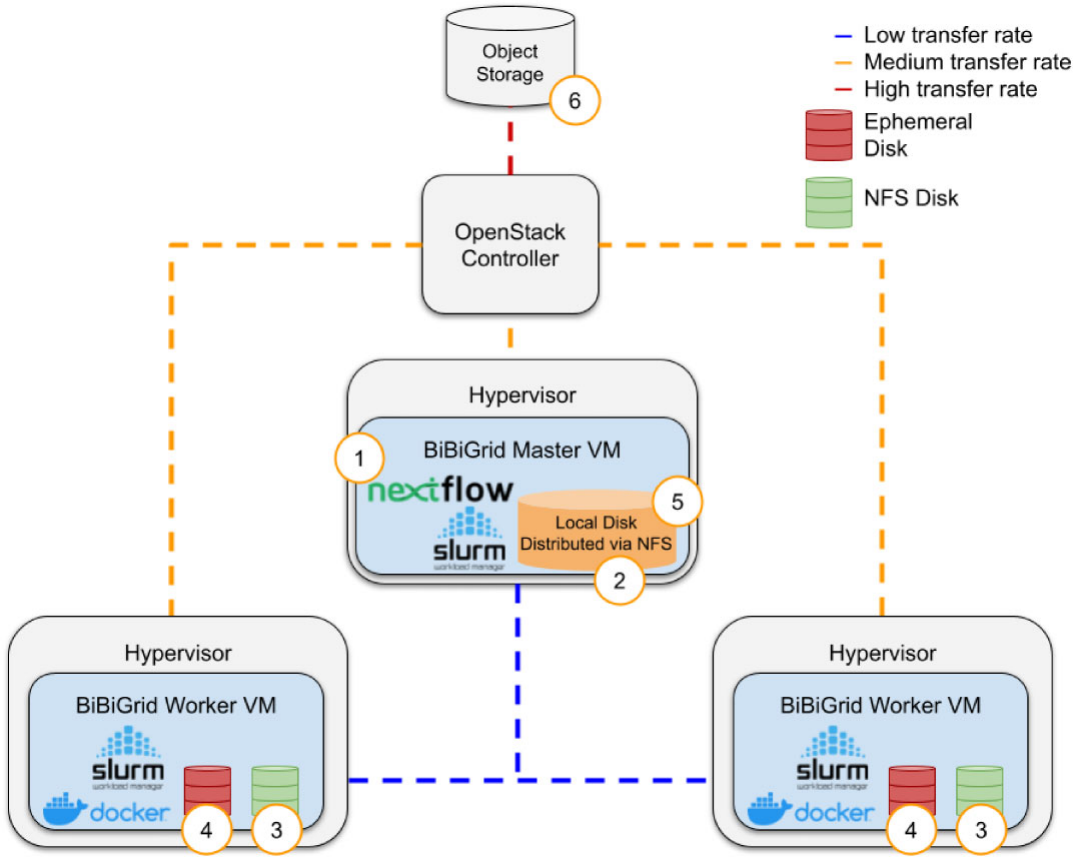
The figure illustrates the data handling of the Metagenomics-Toolkit deployed on a simplified BiBiGrid cluster, comprising one master virtual machine (VM) and two worker VMs, modeled after the characteristics of a de.NBI Cloud site. The numbers represent typical steps in the execution of the Metagenomics-Toolkit. (1) The Master VM is used to start a Nextflow workflow via SLURM. (2, 3) The Worker VM is used to store intermediate workflow results on an NFS. (4) An ephemeral disk is used to store intermediate results of a single command. (5, 6) Results of individual commands are stored on the NFS, while final results are transferred to the object storage.

To perform our analysis using the Metagenomics-Toolkit, we used BiBiGrid, an open source tool for setting up and managing clusters in cloud environments. For our use case, BiBiGrid will set up a cluster with the SLURM workload manager installed. Figure 1 shows a simplified BiBiGrid setup, with a ‘master’ VM used to submit jobs via the Nextflow binary, and two ‘worker’ VMs tasked with executing the submitted workflow commands. Across all VMs, BiBiGrid will also set up a Network File System (NFS)-based shared file system for Nextflow’s working directory, which collects intermediate results. In addition, each worker has a “scratch“ disk, which is located on a hard drive on the host of the respective worker VM.

For our analyses, we leveraged the de.NBI Cloud infrastructure services OpenStack and SimpleVM (https://simplevm.de). Using OpenStack, we configured VMs with customizable settings, known as flavors, to define parameters such as CPU count and RAM capacity. A limitation of the setup shown in Fig. 1 is the shared bandwidth between VMs, which can result in high latency when multiple processes, potentially running on hundreds of VMs, are accessing a shared file system at the same time. This synchronization of data between the master and worker VMs can result in reduced data transfer rates. In comparison, data transfer between a VM host and the OpenStack object storage endpoint provides better connectivity and higher transfer rates. Object storage is used to store input and final output data. To mitigate some of these performance bottlenecks, VM hosts are equipped with local SSD drives as ephemeral disks, which provide the fastest data read and write speeds.

### Preparation, feature selection, and machine learning model assessment for the peak RAM prediction

To optimize the parallelization of the Metagenomics-Toolkit, we applied a machine learning approach to estimate the peak RAM consumption of the MEGAHIT assembly. The resulting product of this approach is a model, i.e. a mathematical representation of the knowledge learned from the data, where the process of feeding data to a machine learning algorithm is called training. In the following subsections, we explain the data preparation and feature selection for training and testing a machine learning model. In a final subsection, we describe the assessment of the model. We provide a pseudo code-like overview of the procedure in Supplementary Table S7.

#### Data preparation for training and testing a machine learning model

MEGAHIT applies a multiple k-mer size strategy, where multiple assemblies based on different k-mer sizes are constructed. Our hypothesis is that diversity and sequencing depth of the biological sample are the main factors that influence MEGAHIT’s memory consumption. To predict the peak memory, 1210 metagenome datasets from environments of varying complexity, i.e. soil, biogas reactors, and nasopharyngeal, were assembled twice, using MEGAHIT’s default and meta-sensitive parameter settings. In the latter case, a wider range of k-mer sizes as compared with the default setting is used, leading to a more accurate and complete assembly, but also to a higher RAM consumption. The peak total amount of memory used by MEGAHIT was monitored by Nextflow and extracted from Nextflow’s trace file.

We applied 10-fold cross-validation for every model, where the training set itself was randomly split in multiple folds consisting of a validation and test subset. Finally, we compared different regression models, then fine-tuned and evaluated the best performing model.

#### Selecting suitable features for the prediction of peak memory

We started by inspecting the following features: number of bases, GC content, minimum read length, average read length, total number of reads, and the k-mer counts of size 13, 21, and 71 of all datasets produced by K-mer Counter (KMC). Based on the resulting k-mer counts, we extracted the following parameters: total number of k-mers, total number of distinct k-mers, mean, standard deviation (SD), maximum of the total number of k-mers per k-mer frequency, and the sum of the lowest 5% (Quantiles 5 k-mers) and highest 5% (Quantiles 95 k-mers) of the total number of k-mers per k-mer frequency. In addition, we included the Nonpareil community diversity index, which summarizes the redundancy or uniqueness of sequences within a given dataset.

To reduce the number of features needed to predict actual peak RAM usage, we examined the Pearson correlation coefficient between the variables and the peak RAM consumption (Supplementary Fig. S1). For generating different machine learning models, we selected 18 features with a Pearson correlation coefficient >0.6 (*P*-value < 0.05), as follows: GC content, Nonpareil diversity index, total number of reads, minimum and average read length, total number of bases, and k-mer counts of size 13, 21, and 71. Based on k-mer counts, we extracted the following features: total number of k-mers (k-mers 13, 21, and 71), total number of distinct k-mers (k-mers 21 and 71), mean (k-mers 21 and 71), SD (k-mers 21 and 71), maximum of total number of k-mers per k-mer frequency (k-mer 13), and sum of the lowest 5% (Quantiles 5 k-mers, k-mers 13) and highest 5% (Quantiles 95 k-mers, k-mers 13, 21, and 71) of the total number of k-mers per k-mer frequency.

#### Assessing machine learning models and reducing the final set of features

We applied several regression-based machine learning methods, namely linear regression, support vector machines, decision trees, voting regressor, Random Forest, and Extremely Randomized Trees. Based on the mean and SD of each root mean square error (RMSE) of the cross-validated sets, we compared the resulting models (Supplementary Table S3). Based on these comparisons, we chose the Extremely Randomized Trees regressor, which had the lowest mean and SD of the RMSE in the default model and the third lowest mean in the meta-sensitive model, respectively, and the lowest SD among the best three models. The main difference between Extremely Randomized Trees and Random Forest is that while both use decision trees, Extremely Randomized Trees use random thresholds based on random features to perform a split in the tree. In addition, the Extremely Randomized Trees regressor allows easy retrieval of feature importances and thereby conducts feature selection. As a next step, we optimized the hyperparameters, using an exhaustive grid search over provided parameters, such as the minimum number of samples at a leaf node.

### Available tools and configuration details for the sewage core microbiome analysis

The Metagenomics-Toolkit offers several tool options within each category (assembly, binning, etc.). For convenience, we provide a list of all available tools in Table 1, and Supplementary Table S4 is a more detailed table, that also highlights tools used specifically for the analysis of the sewage core microbiome analysis. Regarding the core microbiome calculation, we will focus on the mapping configuration part, which is important for the selection of the core microbiome members, while the final configuration file can be found in the Data availability section. We took all Sequence Read Archive (SRA) run accession IDs of all sewage samples from a previous publication [18] and extended them with a region assignment according to World Bank country groupings (Supplementary Fig. S5), similar to the groupings made by Jespersen *et al.* [19].

**Table 1.** List of all tools that are available in the Metagenomics-Toolkit

Modules	Tools
Annotation	MMseqs2 [44], MMSeqs2 taxonomy [42], GTDB-tk [41], CheckM [61], Prokka [40], Prodigal [80], RGI [43]
Assembly	Flye [31], Assembler Resource Estimator, Spades [29], MEGAHIT [30]
Binning	MetaBAT2 [38], MetaCoAG [33], Metabinner [39], MAGScoT [81]
Co-occurrence	Spiec-Easi [63], igraph [64], further R libraries
Dereplication	Pyani [82], SANS [83]
Genome-scale metabolic modeling	CarveMe [67], MEMOTE [69], SMETANA [70], GapSeq [68]
Input	Pysradb [84]
Multiple modules	Minimap2 [36], Bowtie2 [34], Mash Screen [53], Seqkit [85], SAMtools [86], CoverM [37], BWA-MEM [87], BWA-MEM2 [35]
Plasmids	Platon [54], ViralVerify [55], MOB-suite [58], SCAPP [32], PlasClass [56],
	PLSDB [57]
Quality control	Fastp [23], Porechop [27], Filtlong [28], Nanoplot [88], KMC [24], Nonpareil [25]

Tools that are used in multiple modules have the module name “Multiple modules”.

We removed all negative controls from the 951 samples and selected only one sample from each biological replicate. This resulted in 757 sewage samples with an average size of 12.9 Gbp before and 11.6 Gbp after quality control by running the Metagenomics-Toolkit on two de.NBI Cloud sites. After assembly, binning, and dereplication, we mapped each sample against a dereplicated set of 3473 MAGs that are at least 50% complete and are at most 5% contaminated according to CheckM. Completeness and contamination are calculated by counting lineage-specific marker genes. We require that at least 90% of the genome is covered by 1-fold base coverage. Supplementary alignments are excluded, and mapped reads are only accepted if the read is at least 95% aligned with 95% identity. The final abundance table can be inspected in Supplementary Table S5.

## Results

### Metagenomics-Toolkit: an overview of its design and implementation

**Figure 2.**
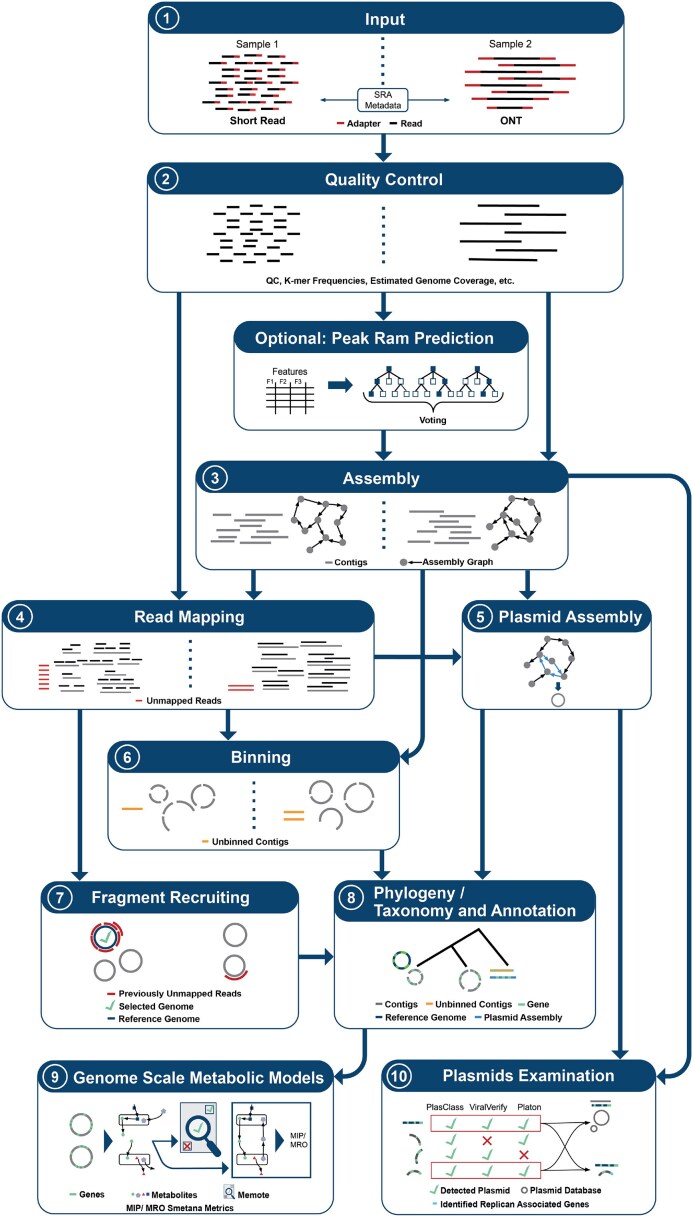
A simplified overview of the Metagenomics-Toolkit single-sample workflow. The processing of different kinds of reads in the main modules of the Metagenomics-Toolkit is illustrated in a step-by-step manner, from top to bottom. The split modules illustrate the different processing methods for short reads on the left and long reads on the right of a module. The metadata of the input reads are checked against the SRA metadata to determine whether ONT or Illumina is provided when SRA is used as a resource for input files (1). All reads are then first quality controlled (2). If Illumina reads are provided and MEGAHIT is selected as the assembly tool, then the peak RAM is predicted. After assembly (3), the contigs are provided as input to the read mapping module (4) and the assembly graph is used for plasmid assembly (5) and optional binning (6). Reads that could not be mapped back to the assembly are mapped against a set of genomes provided by the user (7). All contigs, including the plasmid assembly and genomes detected in the fragment recruitment step, are used as input to the annotation module (8). Predicted proteins are used as input to the genome-scale metabolic modeling module (9). This module creates organism-specific models representing the potential intake and output of metabolites necessary for growth. These models are then checked for quality by Memote, while Smetana metrics are computed. All contigs, and also those from the plasmid assembly, are provided to the plasmid module (10) for further analysis.

**Figure 3.**
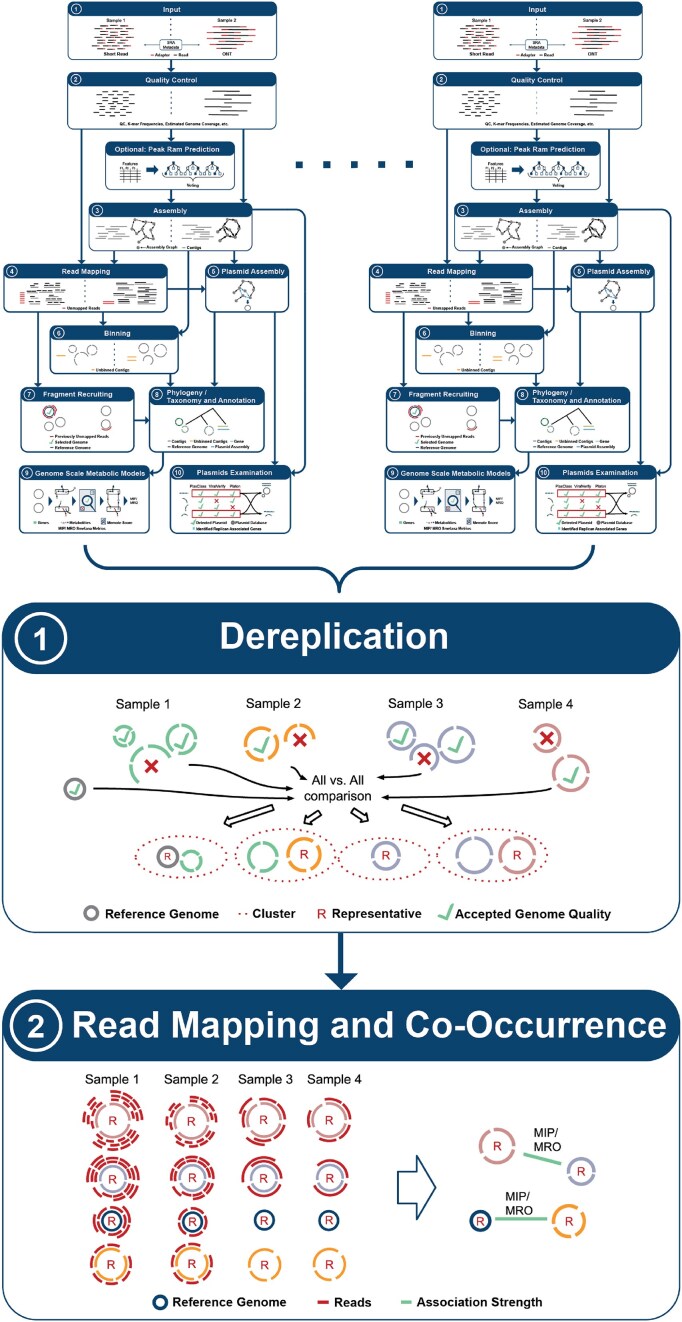
A simplified overview of the aggregation part of the Metagenomics-Toolkit. Once the single-sample workflow has been completed, the aggregation of all datasets is initiated. As illustrated in the figure, the results of single-sample workflows serve as input for the aggregation workflow. Redundant species MAGs are dereplicated (1) in order to obtain a unique set of representative species MAGs. The reads of all samples are mapped against the representative MAGs (2). Based on the abundance values, the co-occurrence of the representative MAGs is calculated. The edges of the resulting co-occurrence network are annotated with the metabolic interaction potential (MIP) and the metabolic resource overlap (MRO) metrics.

The Metagenomics-Toolkit combines several well-established bioinformatics tools and methods by using the workflow system Nextflow to create related modules and combining them in novel ways. A short list of all available tools can be found in Table 1, a more detailed version in Supplementary Table S4, and a summary of all modules and their connections can be found in Figs 2 and 3. Before detailed explanations are given in subsequent sections, this section provides an overview of the Metagenomics-Toolkit modules and their functionality.

In addition to the functionality of running all parts of the workflow consecutively, the Metagenomics-Toolkit is subdivided into so-called modules that can be executed separately. In most cases, modules contain multiple tools for the same or similar type of application. The user can select the appropriate tool for a particular dataset based on personal preferences or the findings of benchmarking projects such as the CAMI challenges [20, 21]. The selected tool will then be used in the entire workflow run or only as part of a chosen module. The workflow accepts a path, link, or S3 address to files containing paired-end or Oxford Nanopore sequences as input.

The Metagenomics-Toolkit automatically converts outputs from each module into formats compatible with the input requirements of subsequent tools. This ensures that once the initial input files for the first tool are provided, all subsequent steps are performed automatically. Users are also not required to start at the beginning of the workflow; instead, they can jump into specific steps by providing the appropriate input files required for those steps.

In general, the Metagenomics-Toolkit follows a two-step strategy for generating MAGs. In a first step, hereafter referred to as the “per-sample” step, all samples are assembled and binned independently, which is expected to result in higher quality genomes compared with a co-assembly approach [22]. Subsequently, in a second step, called aggregation, all MAGs are dereplicated into clusters at the species or strain level [5]. MAGs that have been dereplicated in the second step are analyzed for possible associations using a co-occurrence approach. Both steps can be performed in one or separate calls. The advantage of this step-wise approach is the option to process a large number of samples independently, allowing the use of multiple independent compute infrastructures. The optional aggregation can be done by providing the output of the per-sample step as an input to the second one.

Alongside the technical features outlined in the following sections and the standard functionality commonly found in metagenomics workflows, such as assembly, binning, and annotation, the Metagenomics-Toolkit offers distinctive functionalities. These functionalities serve two particular purposes: first, the automated (re-)analysis of publicly available datasets; and second, the enhancement of the analysis of MAGs, plasmids, and metagenomic datasets as a whole, which will be the focus of the following sections.

#### Reconstruction and annotation of MAGs

The Metagenomics-Toolkit can be executed in either its short- or long-read mode, either automatically determined by evaluating corresponding metadata or manually specified by the user. The choice of the mode affects multiple parts of the workflow as illustrated in Fig. 2 (points 2–7) and Fig. 3 (point 2).

In its short-read mode, raw short reads are subjected to quality control (Fig. 2, point 2) by using Fastp [23] for adapter removal and quality trimming. Additionally, k-mer count frequencies are generated using KMC [24], and the Nonpareil [25] diversity is determined using the tool “Nonpareil”. Both values are necessary for subsequent analysis steps if peak RAM prediction mode is enabled (Fig. 2, optional). In long-read mode (ONT) [26], Porechop [27] is used for the removal of adapter sequences and Filtlong [28] for read trimming.

While in short-read mode, the user can choose to assemble reads using metaSPAdes [29], a short-read assembler, that incorporates sophisticated error correction and assembly graph refinements, enabling high quality, especially in datasets with variable coverage, or MEGAHIT [30], a short-read assembler that uses a multi-k-mer strategy for efficient and accurate assembly of large, complex metagenomes from Illumina data (Fig. 2, point 3). In long-read mode, metaFlye [31], a graph-based long-read assembler that excels at resolving repeats and structural variations in metagenomes, from high error rate sequencing technologies such as Oxford Nanopore or PacBio, is offered. To address variable error rates in Oxford Nanopore sequencing, metaFlye parameters for specifying the expected error rate are automatically determined based on the median PHRED quality score. Resulting assembly graphs are passed for their usage to other modules such as plasmid assembly via SCAPP [32] (Fig. 2, point 5) or binning in MetaCoAG [33] (Fig. 2, point 6).

Pre-processed reads are mapped back to the generated contigs using Bowtie2 [34] or BWA-MEM2 [35] for short reads and Minimap2 [36] for long reads (Fig. 2, point 4). The obtained read coverage information of these mappings is utilized for the next binning step and for the generation of assembly coverage statistics using CoverM [37]. MetaBAT2 [38], a binner that groups contigs into genome bins based on a scoring of tetranucleotide frequencies and abundances, is the default tool for both strategies, but can also be exchanged with Metabinner [39], which groups contigs based on tetranucleotide frequencies, abundances, and a single-copy marker gene analysis, in short-read mode or MetaCoAG. MetaCoAG makes use of the connectivity information found in assembly graphs, composition, coverage information, and single-copy marker genes, along with a graph-matching technique and a label propagation technique to bin contigs, in long-read mode (Fig. 2, point 6).

Prokka [40] predicts and annotates the coding regions of all MAGs, all contigs that could not be binned, and the plasmids that were assembled separately (Fig. 2, point 8). Prokka utilizes a set of pre-defined Metagenomics-Toolkit parameter settings as well as parameter settings that depend on the taxonomic classification of the input sequence. Here, the taxonomy kingdom of the MAG classification using the Genome Taxonomy Database Toolkit (GTDB-Tk) [41], a toolkit for assigning standardized taxonomic classifications to genomes based on the Genome Taxonomy Database, is passed to Prokka for the selection of the correct annotation mode. MMseqs2 taxonomy [42] is used to assign taxonomic labels to all predicted genes based on sequence similarity (Fig. 2, point 8).

Predicted coding sequences are further annotated by the Resistance Gene Identifier (RGI) [43] and MMseqs2 [44] (Fig. 2, point 8). RGI predicts antibiotic resistance genes by conducting a homology search against the Comprehensive Antibiotic Resistance Database (CARD) [43]. As stated in a recent article by Papp and Solymosi, CARD focuses on acquired resistance genes and antimicrobial resistance-associated mutations, and therefore is, for a variety of study settings, the preferable choice in comparison with other antimicrobial resistance gene databases [45].

MMseqs2 [42, 46] is used to search for homologous protein sequences in databases such as KEGG [47] for functional annotation, BacMet [48] for antibacterial biocide or metal resistance, and VFDB [49] for virulence factors. If sufficient memory is available, all databases are stored in RAM to accelerate computations. It is possible to add additional databases according to user requirements. One example is MetaCyc [50], an alternative metabolic pathway database. The user can provide the database as an HTTP/S, S3 link, or a local file path, as long as the database is compressed according to the Zstandard [51]. To accelerate execution of the annotation module, a divide-and-conquer strategy is applied. Large files are divided into smaller chunks, and the annotation is then performed separately on these MAGs, unbinned contigs, and assembled plasmid chunks. This process is carried out in parallel for every chunk and database.

#### Identification of unassembled community members

Current state-of-the-art metagenomic tools are limited in their ability to assemble and bin the entire microbial community, i.e. potentially important genomes may be missed [20, 21]. To address this issue, a fragment recruitment strategy is used to detect known genomes that are part of the community but could not be assembled or binned (Fig. 2, points 4, 6, and 7). First, all reads which failed to map to the assembly are screened against a user-provided database of reference genomes. As this can become computationally time-consuming, if, for example, all representative genomes of the GTDB Taxonomy [52] are used, Mash Screen [53, 35] is run as a fast preliminary filter step. This reduces the possible search space by limiting the number of genomes that have to be checked in the next, more computationally extensive step. Here, identified datasets containing the detected genomes are aligned against the Mash Screen matches using BWA-MEM2 for short reads and Minimap2 for long reads. Finally, alignments are inspected using CoverM. Genomes are reported as a final match if they meet a user-defined percentage threshold of base coverage (default: 90%). In this way, identified reference genomes are then used as additional inputs for all proceeding modules, such as dereplication.

#### Consensus-based plasmid identification

The detection of plasmids within metagenomic datasets can be achieved, in general, by two methods. One approach involves identifying plasmid-specific genes and proteins which can be done via the tools Platon [54] (through the analysis of the replicon distribution differences of protein-coding genes), ViralVerify [55] (based on gene content), and PlasClass [56] (via logistic regression classifiers trained on plasmid and bacterial genome reference sequences). These tools are executed on all contigs of the preceding assembly process. Only contigs for which all specified tools agree are reported as possibly belonging to plasmids in order to increase the precision of plasmid detection (Fig. 2, point 10). Another way to perform plasmid detection is by assembling them using SCAPP, a tool that identifies circular paths in assembly graphs (closed loops) of MEGAHIT, metaSPAdes, or metaFlye as plasmid candidates. Finally, detected plasmids are further analyzed. Possible similar plasmids are searched in the PLSDB [57], to distinguish between previously reported and novel plasmids. Other characteristics, such as the predicted host range of the plasmid, are analyzed using MobTyper [58].

#### Co-occurrence analyses and metabolic modeling

The co-occurrence module, which is part of the aggregation step (Fig. 3, point 2), allows users to analyze co-occurring organisms in different datasets based on their per-sample occurrence and abundance. For example, the node centrality of the co-occurrence network could be examined to see with how many organisms each organism is associated in the network. Node centrality and other methods of network theory such as node clustering can be applied to the resulting co-occurrence networks as part of the downstream analysis, providing valuable insights into the complex structure of microbial communities. In human-related microbiome datasets, co-occurrence allows inference of the influence of co-occurring organisms on the host’s health [59]. Due to the inherent difficulty in interpreting the resulting co-occurrence networks [60], metrics derived from genome-scale metabolic modeling (Fig. 2, point 9) are included in the final co-occurrence network. In the following section, we will first describe the functionality of the independent modules and finally explain the integration of genome-scale metabolic modeling and co-occurrence.

MAGs of different samples that were generated in the per-sample step of the workflow are assigned to species or strain clusters by applying a hierarchical strategy adopted from Pasolli *et al.* [5] (Fig. 3, point 1), which proceeds as follows. First, all generated MAGs are filtered by completeness and contamination, and then pre-clustered via Mash. Mash is employed as a preliminary step that is both fast and efficient in its use of resources. As in the fragment recruitment step, Mash distances of all MAGs were used for an average linkage clustering. Clusters were formed by using a 95% cut-off. In a second step, a representative genome is selected based on a scoring system that considers CheckM [61], a tool for assessing genome completeness and contamination using lineage-specific marker genes, completeness, CheckM contamination, CheckM heterogeneity, N50, and coverage for each cluster. Additionally, a slower but more accurate Average Nucleotide Identity (ANI) computation is conducted between all representatives in order to improve cluster formation. In cases where the ANI of two representatives is >95%, their respective clusters are merged.

Quality-controlled reads from all samples are mapped against all representative genomes to determine the abundance of each genome in each sample (Fig. 3, point 2). Based on the compiled abundance table, possible associations between MAGs (Fig. 3, point 2) can be calculated using two different approaches. The first approach uses pairwise Spearman’s non-parametric rank correlations. Specifically, *P*-values are calculated for correlations between each pair of MAGs for multiple permutations of the abundance table. Adjusted *P*-values are then obtained using the Benjamini–Hochberg procedure [62]. Finally, only reliable associations are used based on the adjusted *P*-values. The second approach is based on co-occurrence networks computed from genome abundances extracted from 16S rRNA gene datasets. Here, Spiec-Easi [63], a tool for constructing microbial association networks based on sparse inverse covariance estimation, is applied on the abundance table to infer an underlying graphical model using the concept of conditional independence. The resulting associations between MAGs, regardless of the chosen approach, represent a co-occurrence graph whose nodes are further annotated by using the GTDB taxonomy. The graph can then be analyzed by using network theory and plotted by Python/R libraries and tools such as igraph [64] or gephi [65].

The Genome-Scale Metabolic Modeling module generates genome-scale metabolic models (GEMs) that are mathematical representations of chemical reactions within a microbial organism [66]. GEMs are generated from the corresponding annotation of all high-quality MAGs via CarveMe [67] or GapSeq [68], and quality controlled using a tool, for standardized testing and quality control of genome-scale metabolic models, called MEMOTE [69] (Fig. 2, point 9). Further possible cases of cross-feeding and competition between microbiome members per sample are assessed by running SMETANA [70]. SMETANA outputs metrics such as the metabolic interaction potential (MIP) and the metabolic resource overlap (MRO). Depending on the available amount of computational resources, the user can enable the computation of the species coupling score (SCS), which measures the dependency of the growth of a given species in the presence of another species in a community. MRO, MIP, and SCS allow the researcher to make assumptions about the degree of a possible interaction between microbial community members.

The co-occurrence network and metrics derived from genome-scale metabolic modeling are combined by computing the MIP and MRO metrics for each pair of MAGs connected by an edge. This information can be used to analyze the reason for their association. A co-occurring pair of MAGs with a high MIP could indicate cross-feeding, while a high MRO value could indicate that organisms are competing for the same resources.

### Optimizing efficiency for cloud-based cluster system

While it is possible to run the Metagenomics-Toolkit on a single-user workstation, the key design decision for the Metagenomics-Toolkit was to use cloud-based compute clusters that allow workflows to scale in cloud environments using proven job workload managers such as SLURM. Figure 1 illustrates an exemplary setup that we have employed using the BiBiGrid open source tool for a cluster configuration inside a cloud environment. The following sections describe the optimizations that have been implemented to make the Metagenomics-Toolkit particularly compatible with cloud-based cluster environments. The characteristics and possible challenges of a cloud site are described in the Materials and methods and visualized in Fig. 1. In essence, due to bandwidth limitations, software should reduce the data transfer between VMs and instead use the local disk and object storage for input, output, and intermediate results.

#### Efficient and automated input data and database download

The Metagenomics-Toolkit enables automated and streamlined processing of publicly available datasets by specifying a list of SRA or study IDs that are automatically retrieved either directly from NCBI or from a user-defined mirror. Individual datasets can be processed by providing a list of sample names and paths to their respective locations, either locally, by HTTP/S, or by S3. Prior to any dataset download, the correctness and accessibility of SRA paths are verified, along with any associated metadata for public datasets, which are automatically downloaded.

In the default execution of Nextflow runs, all input files are stored on the NFS with all intermediate results (Fig. 1, points 2 and 3). This can lead to bottlenecks if too many read/write operations occur at the same time. This can become even more problematic when large sequence databases, such as NCBI-nr, are downloaded to the NFS and queries to these centrally provided databases are distributed across multiple compute instances. To reduce the load on the NFS, the workflow has been optimized to handle file downloads more efficiently. In contrast to the default execution of Nextflow runs, the input fasta files are downloaded directly to the scratch disk of the cluster instance that requires them (Fig. 1, point 4). This also reduces the need for large amounts of space on the NFS. Keeping local copies of reference databases on the scratch disk in each instance is more efficient in many cloud setups. To ensure consistency between all local copies, the Metagenomics-Toolkit integrates an automated task for this purpose. Databases can be uploaded to an S3-compatible object storage and referenced in the configuration of every tool, respectively, in the form of an S3 path and database-specific md5sum hash. During the execution of a tool, required databases are searched in a pre-defined directory that is located on the scratch disk, and its md5sum hashes are checked. If the database is not found or the hashes do not match, i.e. the database is missing or present in a different version, it will be automatically downloaded from the S3 cloud storage and stored on the scratch disk. This ensures that the configured version of the database is always available for analysis. The Unix flock locking process is used to ensure that multiple downloads of the same database are not started at the same time on the same worker node, since each database download is a separate job in the context of SLURM (see the Materials and methods). The Metagenomics-Toolkit can access a large number of databases that are relevant for metagenomic analyses and have been uploaded to the de.NBI Cloud Bielefeld object storage. Corresponding links and checksums are provided in the default Metagenomics-Toolkit configuration file (see Data availability).

#### Tool execution on cloud instances

For tool execution, the Metagenomics-Toolkit utilizes containerization with Docker containers and thus allows for the convenient distribution and execution of software along the worker instances of a cluster without the need for manual installation. When available, we used public Docker images created by the Bioconda community [71], otherwise we created our own (Supplementary Table S4). To avoid high input/output (I/O) activity on the shared NFS which can lead to a high latency, each tool is executed in the scratch directories of the cloud instances. Only results are copied from the scratch directories to the shared NFS. An additional mode allows further reduction in the NFS usage, by circumventing the normal Nextflow SRA input download mechanism, with all remote files being placed directly into the worker scratch directory instead of the usual Nextflow working directory. By using this mode, the raw files do not consume additional disk space on the shared file system and the download can be performed in parallel on the worker nodes.

#### Documentation and visualization

To ensure comprehensive documentation of our workflow, i.e. the structured storage of the workflow execution details, the workflow stores each command, as well as results, in directories named in accordance with the module name, module version, executed tool, and its version number (Supplementary Fig. S4). In conjunction with Docker-based containerization, this approach ensures reproducibility.

Standardizing the output of multiple modules simplifies the use of the Metagenomics-Toolkit as part of other workflows and allows the output of the per-sample step to be reused as input for the aggregation step. Standardization in both cases is particularly useful when tools of the same module are being replaced.

The Metagenomics-Toolkit output can be transformed into an EMGB-compatible input by running a post-processing script on the Metagenomics-Toolkit output files, to create json files containing assembly, binning, and annotation information. EMGB is a web interface that can be used for the visual exploration of metagenomic datasets. Large datasets containing millions of genes and their annotations are pre-processed and visualized, to be searched in real-time by the user. The platform provides access to different aspects of one or more datasets via an interactive taxonomic tree and dynamic KEGG metabolic maps for each dataset, allowing researchers to explore their datasets at the level of genes, contigs, MAGs, pathways, or biological process statistics (Supplementary Fig. S6).

### Machine learning-guided peak memory prediction

By estimating the peak memory consumption of bioinformatics tools prior to execution, it is possible to adjust the resources requested from an infrastructure in order to minimize the resources needed for each job. This approach can speed up the entire workflow by allowing more tools to run in parallel and the optimal use of the allocated cloud resources. Overall costs can be effectively utilized and even reduced by requesting only those resources which are actually necessary. As part of the Metagenomics-Toolkit, we use a machine learning approach (see the Materials and methods for details) to improve the resource specification for two parameter sets of the MEGAHIT metagenome assembler (Fig. 2, point 3). While the Metagenomics-Toolkit allows the assembler to be restarted on error with a user-specified higher amount of RAM, this should only be the last resort. Instead, the main goal is to predict the peak RAM usage as accurately as possible and to avoid out-of-memory crashes while using only the minimal amount of necessary RAM.

Metagenome assembly requires machines with hundreds of gigabytes of shared memory, even for modestly sized datasets [72]. However, RAM consumption varies for different datasets, and optimizing the resource requirements of a metagenome assembler would, at best, obviate the need for dedicated rarely available high-memory hardware. Here, we train a machine learning algorithm that, for simplicity, uses the same features for two different assembler parameter settings.

We assembled 1210 metagenome datasets from environments of varying complexity, i.e. soil, biogas reactors, and nasopharyngeal, twice using MEGAHIT’s default and meta-sensitive parameter settings (Supplementary Table S6). We selected 18 features of the datasets that had a Pearson correlation coefficient of at least 0.6 (*P*-value <0.05) with the reported peak RAM value (Supplementary Table S8) as input for six regression-based machine learning algorithms. The best performing regressor according to our evaluation which was based on cross-validation (see the Materials and methods for details) is the Extremely Randomized Trees regressor (Supplementary Table S3).

After optimizing hyperparameters of the best estimator, we examined the feature importance reported by the regressor (Supplementary Figs S2, S3). Based on the feature importance, we selected the number of distinct k-mers with k = 21 and k = 71 as features for the final model. The Nonpareil diversity index and GC content are also included in the final model but are not relevant according to feature importances and will be removed in future releases. In addition, the Quantiles 5 k-mers number tracks the total number of rare sequences in the dataset. The number of distinct k-mers is influenced by the diversity of the microbial community. The peak memory value depends on the complexity of the de Bruijn graph. This complexity increases with a higher number of distinct k-mers. Finally, we evaluated the best performing estimator of both models by calculating the confidence interval (95%) based on the prediction on the test dataset. The estimator of the default model has a generalization error between 3 and 5 GB RAM, while the meta-sensitive model has an error between 5 and 12 GB RAM. The maximum error reported by the confidence interval is added as a bias to the predicted value. Depending on this value, a VM flavor with the next higher memory value will be set for the execution of this dataset’s MEGAHIT assembly. To examine the error, we calculated the Pearson correlation coefficient between the prediction error and all features of the test dataset. Supplementary Fig. S7 shows all coefficients. The highest correlation observed is 0.47 with the variable “distinct number of k-mers (k-mer size 21)”.

**Figure 4.**
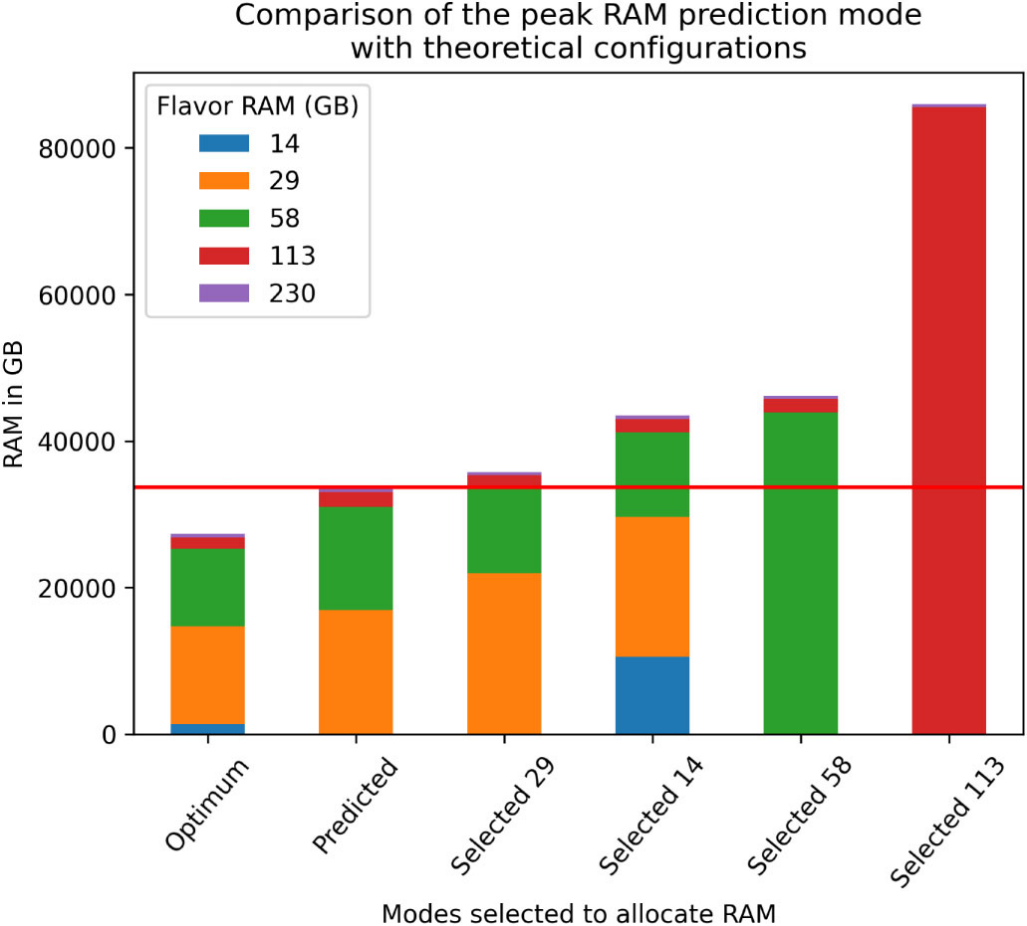
Different RAM configurations when assembling 757 sewage samples. Selected* configurations are theoretical settings, where a user has selected a flavor for all datasets and the workflow only increases the flavor if the assembler fails due to insufficient RAM. The Optimum mode uses the most appropriate flavor for every dataset. The Predicted mode uses the predicted flavor for the respective sample. All modes are calculated using the peak RAM value reported by Nextflow and the predicted peak RAM value for all samples. The total peak RAM value of the predicted mode is represented by a red line. The various flavors used in the BiBiGrid cluster setup and their corresponding RAM sizes are indicated by the different colors.

We tested the peak RAM prediction by processing 757 sewage samples. The tools utilized for the analyses can be inspected in Supplementary Table S4. As part of the assembly module, we assembled these 757 sewage samples after quality control using MEGAHIT’s meta-sensitive parameter setting. Comparing the predicted peak RAM consumption reported by Nextflow, we get a mean error of 7 GB with an SD of 8 GB. In the following, we compare the amount of RAM predicted by an optimal selection of the five flavors used in our BiBiGrid cluster setup and four theoretical approaches (Fig. 4). A scenario in which all assembler runs are started with a specific flavor is defined by the selected* modes (see different selected modes in Fig. 4). In the case of insufficient RAM, the assembly process runs out of memory and the flavor with the next highest RAM value is selected for the next attempt. This procedure continues until the specific sample is assembled. The optimum mode represents the amount of RAM required to assemble all samples on the first try. The theoretical approach that allocates 113 GB of RAM on the initial attempt is the one with the highest RAM consumption in total, while our prediction mode results in the lowest RAM consumption compared with all naive selection approaches. In detail, our approach saves 9736 GB (Selected 14), 2038 GB (Selected 29), 12 449 GB (Selected 58), and 52 276 GB (Selected 113) of RAM.

### Feature comparison of existing metagenomics workflows

The Metagenomics-Toolkit offers different tools, functionalities, and analyses combined with different sequence data input types. While some capabilities are already available in existing workflows, we want to highlight the novel ones in this section. We compared the Metagenomics-Toolkit with five metagenomics workflows, namely MetaGEM, MetaWrap, MUFFIN, SqueezeMeta, and nf-core/MAG, that meet the requirements that the workflow must be fully publicly available, represent a workflow where all parts can be executed in a single call, and be in common use at the time of writing. We compared all workflows in terms of their implemented features in four categories: “Sequence Data Input”, “Analysis Options”, “Data Handling”, and “Other Features”. We only refer to two types of features: one type are features that are implemented by at most two out of five workflows (“Novel Functionality”) and the other type represents features implemented by at least three out of five workflows but not by the Metagenomics-Toolkit (“Missing Features”) (Table 2). A comparison table with all features can be found in Supplementary Table S2.

**Table 2.** Feature comparison between the five workflows MetaGEM, MetaWrap, MUFFIN, SqueezeMeta, and nf-core/MAG

Features	Implemented by MGTK	No. of WFs per feature	MGTK features implemented by at most 2 of 5 WFs	Non-MGTK features implemented by at least 3 of 5 WFs
**Sequence data input**				
Assembly	✗	3		Missed
Single end reads	✗	3		Missed
Long read (Nanopore)	$\checkmark$	2	$\checkmark$	
**Analysis options**				
Co-assembly	✗	3		Missed
Dereplication	$\checkmark$	0	$\checkmark$	
Plasmid detection	$\checkmark$	0	$\checkmark$	
Genome-scale metabolic modeling	$\checkmark$	1	$\checkmark$	
Fragment recruitment	$\checkmark$	0	$\checkmark$	
Pathway annotation	$\checkmark$	2	$\checkmark$	
Antibiotic resistance	$\checkmark$	0	$\checkmark$	
Contig/gene-based taxonomy	$\checkmark$	2	$\checkmark$	
Co-occurrence	$\checkmark$	0	$\checkmark$	
Process unbinned contigs	$\checkmark$	2	$\checkmark$	
**Data handling**				
Aggregate samples as a separate step	$\checkmark$	0	$\checkmark$	
Optimized database management^a^	$\checkmark$	0	$\checkmark$	
S3 usage	$\checkmark$	2	$\checkmark$	
Database extensibility^2^	$\checkmark$	1	$\checkmark$	
**Other functionality**				
SRA processing	$\checkmark$	0	$\checkmark$	
ML-guided parameter estimation	$\checkmark$	0	$\checkmark$	
Allow separate workflows to call	$\checkmark$	2	$\checkmark$	
HPC suited	✗	5		Missed
CI tested	$\checkmark$	2	$\checkmark$	
Visualization platform	$\checkmark$	2	$\checkmark$	

The features listed are either Metagenomics-Toolkit features that are implemented by at most 2 of the 5 workflows, or features that are not implemented by the Metagenomics-Toolkit but are implemented by at least 3 of the other 5 workflows. The abbreviations MGTK and WF refer to the Metagenomics-Toolkit and workflow, respectively. A comparison table with all features can be found in Supplementary Table S2. This table lists features implemented by various workflows and their comparison.

^a^ DB Download, DB integrity check, DB stored in scratch dir.

^b^ User can provide databases.

Novel features available in the Metagenomics-Toolkit are, as described in the previous main section, the co-occurrence, fragment recruitment, plasmid detection, and peak RAM consumption prediction. In contrast to most other workflows, the Metagenomics-Toolkit allows the input of datasets obtained by the Oxford Nanopore sequencing technology. However, it does not calculate co-assemblies and hybrid assemblies where short and long reads are accepted. The Metagenomics-Toolkit offers many types of analysis methods such as a search for genes predicted to mediate antibiotic resistance and genome-scale metabolic modeling of the reconstructed MAGs. In addition, the Metagenomics-Toolkit is optimized to work directly with SRA data and to scale on cloud-based clusters. Finally, a distinguishing feature is the ability to explore metagenomic data via an interactive website with the EMGB platform.

### Global occurrence of species revealed by analyzing members of the sewage core microbiome

This section presents a use case demonstrating the capabilities of the Metagenomics-Toolkit, with a particular focus on the identification of a sewage core microbiome.

After running the per-sample workflow step, which includes assembling and binning of the 757 sewage datasets, we dereplicated all MAGs at the species level and mapped the sequence reads of each sample against the representative genomes. All results of the per-sample step are available as EMGB input files for further investigation (see Data availability). Dereplication of all generated MAGs resulted in a set of 3473 MAGs that are at least 50% complete and at most 5% contaminated. We define the sewage core microbiome as a set of MAGs where each MAG represents a species that is present in multiple samples. Specifically, we are interested in MAGs that are present either in > 60% of all sewage samples or in 90% of all sewage samples from a particular region and their abundance.

**Figure 5.**
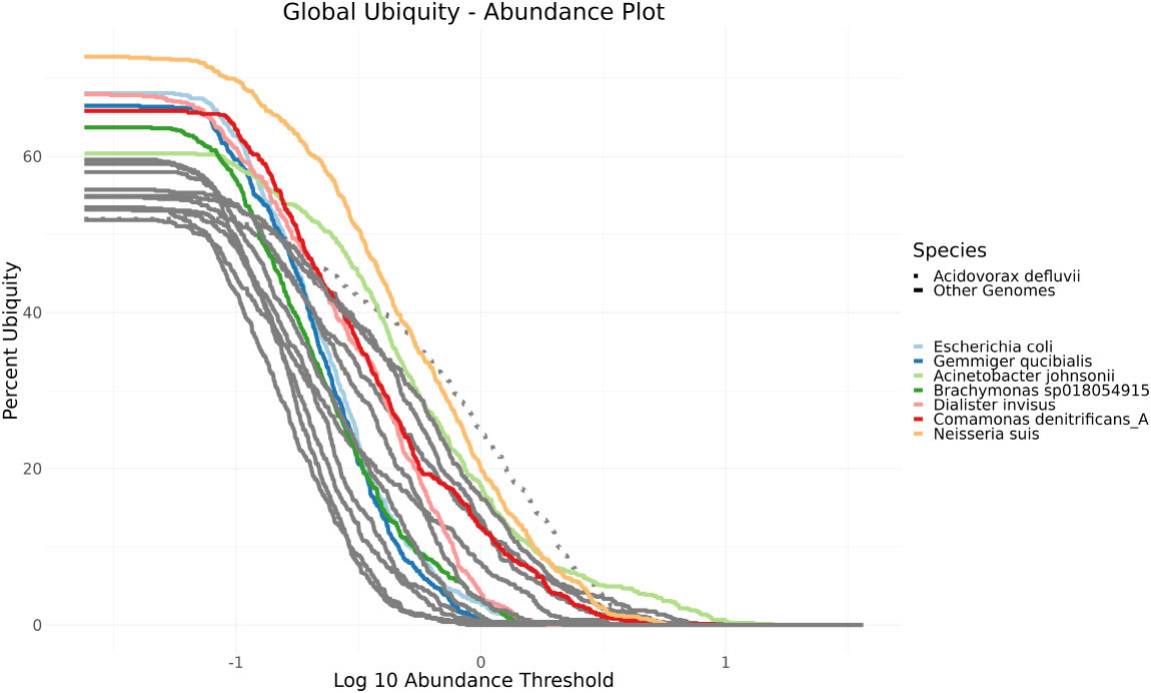
The ubiquity–abundance plot shows for each species its occurrence in samples (Percent ubiquity *y*-axis) with a specific minimum abundance (Log10 abundance threshold *x*-axis). Only species with at least 50% ubiquity are displayed, and species with >60% ubiquity are colored.

For all subsequent analyses, we filtered out datasets below the Q1–1.5 × IQR of the Nonpareil genome coverage percentage, which resulted in the removal of five samples with low sequencing depth according to Nonpareil. We give a general overview of the organisms that meet the aforementioned core microbiome criteria by considering their occurrence dependent on their abundance using ubiquity–abundance plots [73] (Fig. 5). The first insight is that there is no MAG that could be found in all samples. This could also be due to low sequencing depth or technical limitations in the assembly or binning procedures. The MAGs present in >60% of all samples belong to 10 species. Based on the literature (see Supplementary Table S1, column links for details), all species were found in samples from either sewage or wastewater treatment plants, with the exception of *Dialister invisus*, which was isolated from human oral cavity samples.

**Figure 6.**
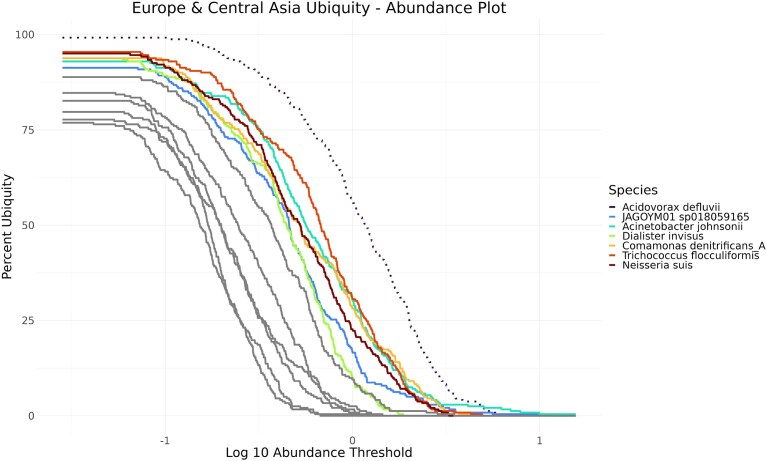
The ubiquity–abundance plot shows for each species its occurrence in samples (Percent ubiquity *y*-axis) with a specific minimum abundance (Log10 abundance threshold *x*-axis). Only species with at least 50% ubiquity are displayed, and species with >60% ubiquity are colored.

In Fig. 5, it can be observed that the curves can cross. This occurs, for example, when a high-ubiquity, low-abundance species is compared with a low-ubiquity, high-abundance species in a subset of samples. One example is the species *Acinetobacter defluvii*, represented by a dotted line, as the species with the highest abundance in 11.3% of all samples. *A. defluvii* is particularly common in samples taken in Europe and Central Asia (Fig. 6), where it occurs in 99.1% of all samples and is the most abundant species in 29.7% of all datasets. Another continent where *A. defluvii* occurs in many samples is North America, with 83.5%. In 13.9% of all North American samples this species featured the highest abundance.

Considering the regional differences, regional core microbiomes on a continental scale were examined based solely on ubiquity. Here we applied an approach similar to the “range-through” approach described by Neu *et al.* [74]. We search for species that are present in >90% of all samples from a country and only include these species in the next step. Only if a species could be detected in 80% of all available countries in a region do we define it as part of the core microbiome of that region. Using this strategy, the following possible members of a core microbiome were detected (Table 3). *Neisseria suis* in combination with *A. defluvii* are members of the core microbiome of sewage samples in the Europe and Central Asia region, and *D. invisus* is a core microbiome member in North America.

**Table 3.** List of organisms that are present in >90% of all samples from a country and their percentage ubiquity in the country grouping according to the World Bank

Country grouping	Species name	Percentage of countries	Checkm	Checkm
according to World Bank	according to GTDB	of a World Bank grouping	completeness	contamination
		where the organism	(%)	(%)
		could be detected		
Europe and Central Asia	*Acidovorax defluvii*	94.87	74.95	2.39
	*Neisseria suis*	82.05	77.59	0
North America	*Dialister invisus*	100	65.52	0
Sub-Saharan Africa	*Neisseria suis*	45.45	77.59	0
Middle East and North Africa	*Desulfobulbus sp017998195*	71.42	98.81	0
	*Comamonas denitrificans*_A	71.42	91.71	2.27
	*JAGPMH01 sp018052945*	71.42	99.28	0.63
	*Neisseria suis*	71.42	77.59	0
East Asia and Pacific	*Escherichia coli*	38.46	98.57	0.94
Latin America and Caribbean	*Neisseria suis*	53.84	77.59	0
South Asia	*Escherichia coli*	60	98.57	0.94

Only species with the highest ubiquity per grouping or with a ubiquity >80% are listed.

In Latin America and the Caribbean, South Asia, the Middle East and North Africa, sub-Saharan Africa and East Asia, and the Pacific region, the most ubiquitous species occurs in 53.84, 60, 71.42, 45.45, and 38.46% of all countries, respectively (Table 3).

## Discussion

In this work, we presented the Metagenomics-Toolkit, a scalable workflow that provides fully reproducible results, offers different analysis methods, and can process datasets on single workstations, but is particularly capable of handling large amounts of data in SLURM-based clusters hosted on cloud infrastructures. The division of the workflow into a per-sample and an aggregation step allows the distribution of the first part to multiple cloud sites to complete the analysis in an acceptable time frame, especially in demanding cases with thousands of samples.

To reduce the likelihood of programming errors being introduced, the Metagenomics-Toolkit is tested regularly against simulated datasets and real-world datasets by using Github Actions. These tests are executed for each module and a range of different combinations of modules.

While we have optimized the input and database handling, the overall performance can be further improved by relying less on a shared file system and more on object storage. Which solution Nextflow offers to move the working directory to an S3-compatible object storage must be further investigated.

Our approach reduces the need to request valuable, rarely available high-memory hardware. At the time of writing, the price difference between a VM with 64 GB of RAM (c5a.8xlarge, US$1.392 per hour) and a VM with 256 GB of RAM (r5b.8xlarge, US$2.848 per hour) is over twice as much. Moreover, we ran the workflow on a cloud where high-memory node availability is limited. Therefore, the overall execution benefits from fewer requests for high-memory nodes.

It should be noted that in addition to the benefit of our prediction mode enabling the lowest RAM consumption in comparison with the naive approaches, it automatically selects the VM flavor with the next higher RAM value in the case of an error. Combined with error handling, the prediction mode eliminates the need for manual intervention and thereby automates the failover processing of a large number of datasets. Although in our theoretical modes, the selected medium flavor mode is close to our prediction mode, in practice, the user would have to estimate the actual RAM consumption based on experience. The error in selecting the wrong flavor based on an inaccurate predicted peak RAM consumption depends on the user-defined flavor set. Setting wider RAM flavor boundaries leads to more correct flavor selections. An additional insight is that according to the feature importances (Supplementary Figs S2, S3) of both models, the size of the dataset (number of bases) is not as important as the k-mer statistics for a correct estimation. Regarding the error, we could not find a common characteristic across the datasets where predicting the peak RAM resulted in a larger error. We assume two possible reasons that are open to future investigation. One reason could be that there is another variable that we have not yet found, while another reason could be that the chosen machine learning algorithms are not able to correctly incorporate or weight the chosen variables (see Supplementary Fig. S6). It is to be noted that due to the bias that is added to the predicted value, low-complexity datasets that may only require a few gigabytes of RAM will also always end up with an additional gigabyte of RAM according to the bias. This may make it impossible to run the assembly on a user workstation, i.e. a laptop. In these cases, the prediction mode can be disabled in the configuration file. While the created machine learning model only applies to MEGAHIT, the machine learning method we used to create the model allows it to be applied to other assemblers as well, such as metaSPAdes. In addition, it needs to be investigated whether the model can be further simplified without loss of accuracy. Another point worth investigating is the workflow execution time saved by using peak RAM prediction. The number of CPUs and the amount of RAM are interdependent and, since our method tries to reduce the amount of RAM, it also reduces the number of CPUs. Otherwise, our approach reduces the number of times the assembly has to be retried.

Regarding the use case being performed, to our knowledge it is the first analysis of the sewage core microbiome with such a large number of datasets. Concerning the core microbiome definition, it was pointed out by Neu *et al.* [74] that there is no single ubiquity or abundance threshold that has been used in previous studies to define a core microbiome. Therefore, we utilized ubiquity–abundance plots to give the reader an overview of a range of possible thresholds before we defined our own ubiquity threshold. However, we only used ubiquity thresholds, since low-abundance genomes could also be important members of a community and we do not have any reason to exclude them when defining the core microbiome. Our analysis highlights the distribution of species worldwide and in specific regions. We examined not only the presence but also the abundance of all organisms, allowing future work to analyze the potential spread of antibiotic resistance genes and associated microorganisms such as *Escherichia coli*.

In general, we expect that members of the core microbiome are widespread organisms that have characteristics such as versatility in terms of nutrient uptake, stress tolerance, or adaptation of defense mechanisms, and to be able to withstand seasonal effects, as samples were taken at different times of the year. One of the organisms we detected is *Acinetobacter johnsonii* which, according to Jia *et al.* [75], shows exceptional adaptability to occupy different environments. However, the actual characteristics of the organisms should be investigated in follow-up analyses. It should also be noted that although we present a geographical distribution of the organisms, it needs to be investigated whether the occurrence of the organisms depends on geography or on other factors.

We have shown that the Metagenomics-Toolkit is ideal for large-scale and efficient analysis on cluster-based cloud systems, enabling the investigation of the sewage core microbiome by processing 757 metagenome samples. The uniqueness of its feature set is discussed further in the following.

Comparison of the Metagenomics-Toolkit with other workflows showed that several other workflows offer co-assembly functionality, which is particularly useful for recovering MAGs with low sequence coverage. However, there is a trade-off between our implemented separate assembly combined with a dereplication approach and co-assembly. Co-assembly is expected to generate MAGs with higher completeness, and to yield more low-coverage genomes. However, it results in higher contamination compared with single assemblies and subsequent dereplication [76]. It remains to be evaluated in which cases co-assembly should be preferred over our approach or in which cases a combination of both strategies could be used.

In addition to the co-assembly functionality, future enhancements will focus on the improvement of specific modules and on additional input types such as assembled contigs, transcriptome data, and a combination of short and long reads for hybrid assemblies. The co-occurrence module can be better integrated with genome-scale metabolic modeling. One example would be that detected subcommunities in the network could be better investigated according to their MRO and MIP values. In general, new modules can be introduced that provide a pangenome analysis or the analysis of viral genomes.

Finally, due to the extensive use of Docker, the Metagenomics-Toolkit needs to be adapted for use in HPC environments. Nextflow allows one to easily specify other HPC-friendly container engines such as Singularity [77], Apptainer [78], or Podman [79].

## Supplementary Material

lqaf093_Supplemental_Files

## Data Availability

All tools and containers that were used can be viewed by accessing the tag “0.3.0-rc.15” of the https://github.com/metagenomics/metagenomics-tkrepository. The code has also been archived on Zenodo at https://doi.org/10.5281/zenodo.14989604. The actual configuration with a list of used databases can be found in this repository: https://github.com/metagenomics/wastewater-study The Metagenomics-Toolkit output and EMGB inputs of all sewage datasets are publicly available via the S3 link s3://mgtk/data/ using the endpoint URL https://s3.bi.denbi.de. Further details can be found in the associated dataset https://doi.org/10.26165/JUELICH-DATA/KXDWII.
