# Supplementary material for: Metagenomics-Toolkit: the flexible and efficient cloud-based metagenomics workflow featuring machine learning-enabled resource allocation
Source: NAR Genom Bioinform. 2025 Jul 17;7(3):lqaf093. doi: 10.1093/nargab/lqaf093 (PMC12267984; doi:10.1093/nargab/lqaf093)
Supplement: lqaf093_Supplemental_Files [file lqaf093_supplemental_files.zip › supplementary_figures_R2.pdf]

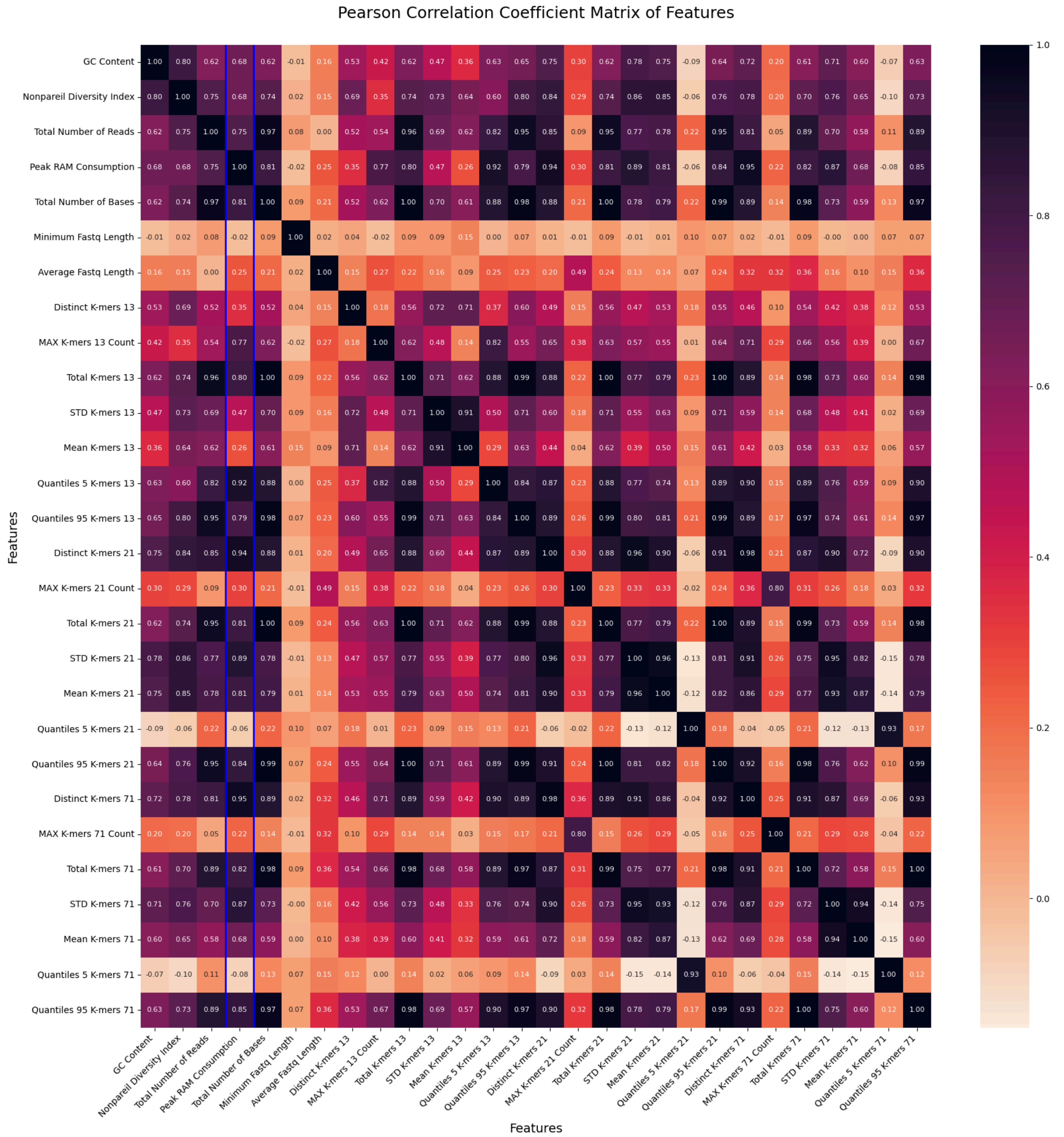

Supplementary Figure 1: Pearson correlation matrix of possible features for a machine learning algorithm.

Explanation

- MAX K-mer * Count: The maximum of the total number of k-mers per k-mer frequency.
- Total K-mers *: The total number of k-mers.
- STD K-mers *: Standard deviation of total number of k-mers per k-mer frequency.
- Mean K-mers *: Mean of the total number of k-mers per k-mer frequency.
- Distinct K-mers *: Number of distinct k-mers.

- Quantiles 5 K-mers *: The sum of the lowest 5% of the total number of k-mers per k-mer frequency.
- Quantiles 95 K-mers *: The sum of the highest 5% of the total number of k-mers per k-mer frequency.

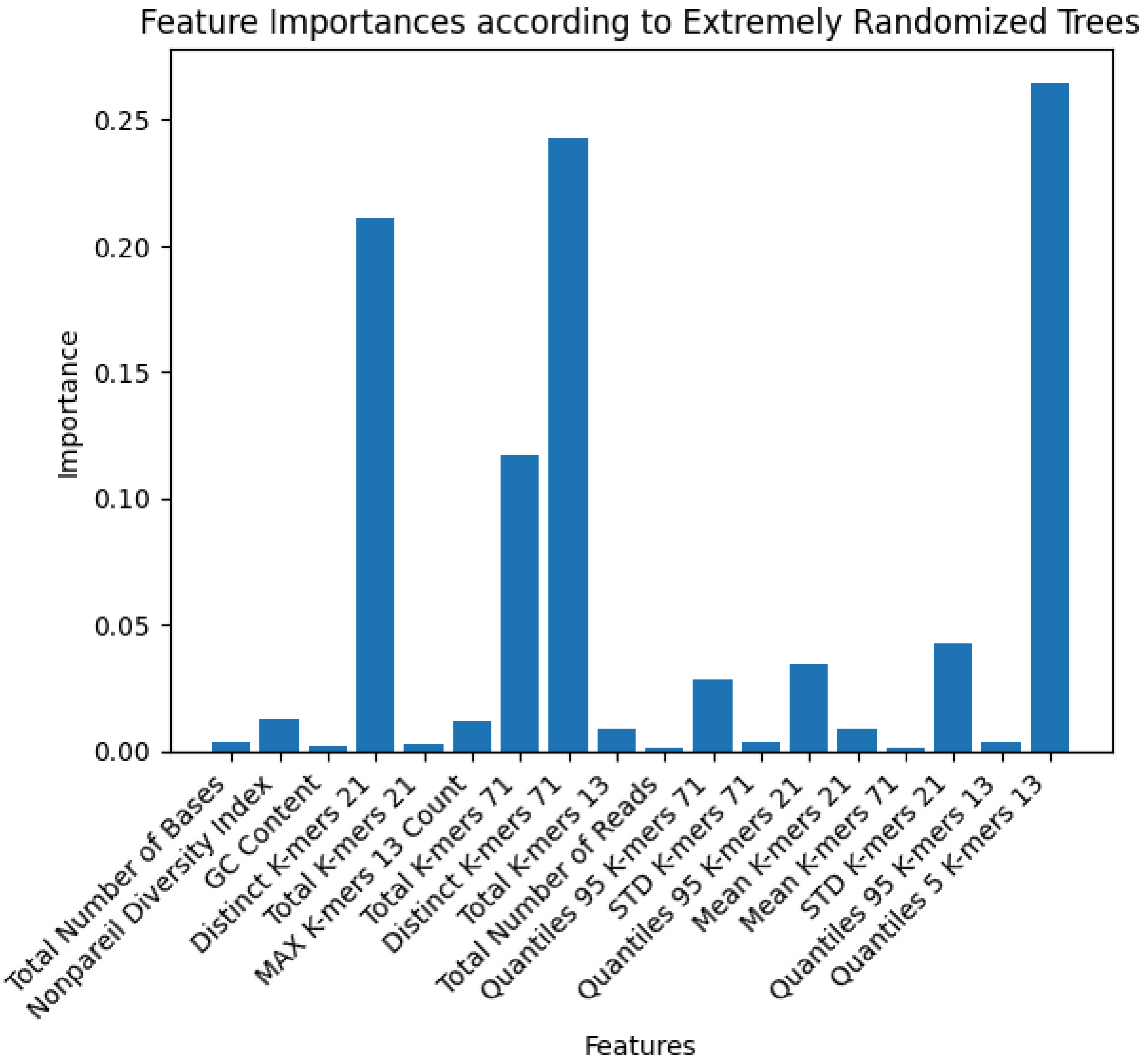

Supplementary Figure 2: Feature importances according to Extremely Randomized Tree approach based on Megahits default parameters.

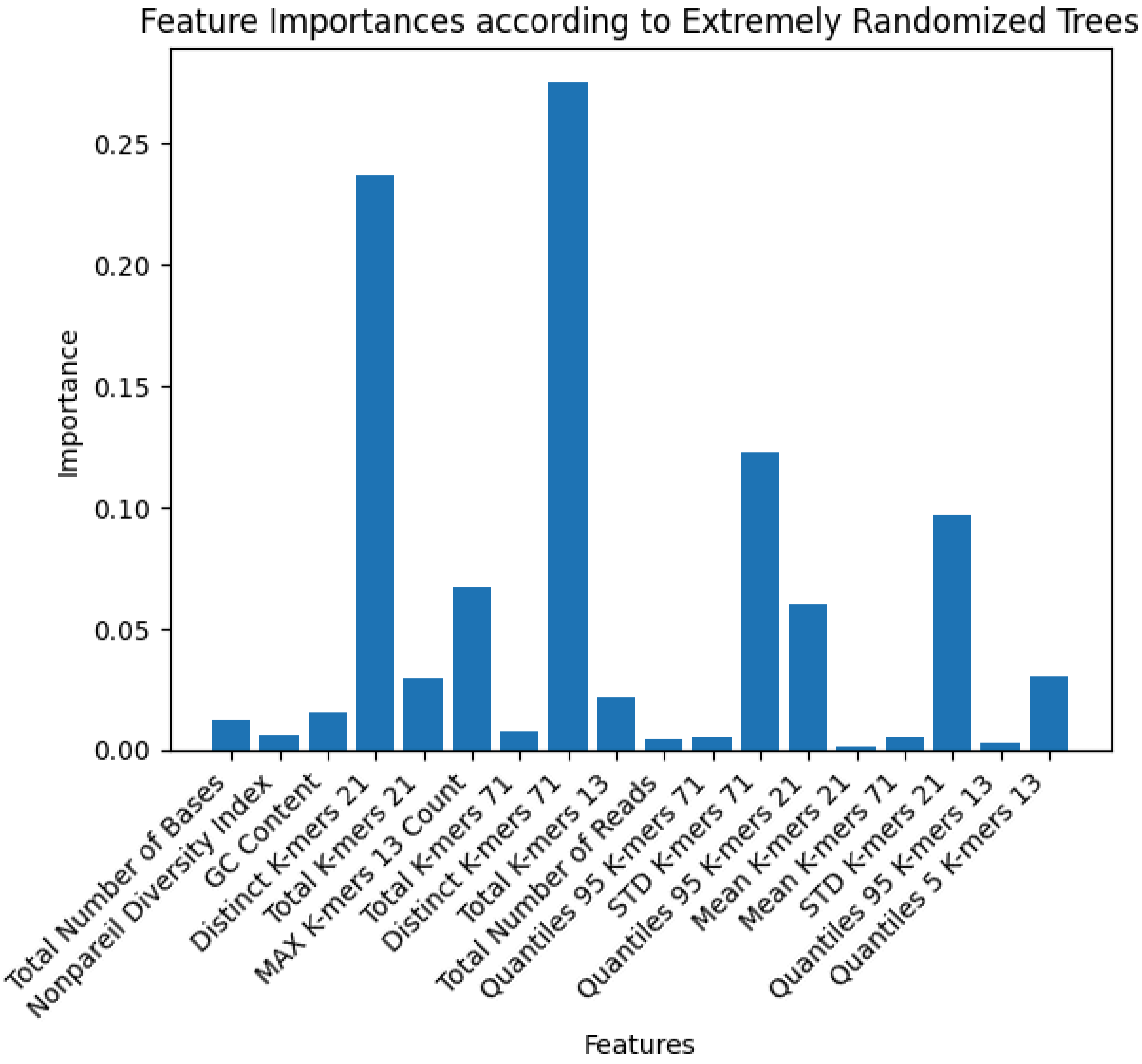

Supplementary Figure 3: Feature importances according to Extremely Randomized Tree approach based on Megahits meta-sensitive parameters.

```
AGGREGATED
└── 1
    └── fragmentRecruitment
        └── 0.1.0
            └── mash
                └── paste

ERR4678561
└── 1
    ├── annotation
    │   └── 0.3.0
    │       ├── mmseqs2
    │       │   ├── bacmet20_experimental
    │       │   ├── bacmet20_predicted
    │       │   ├── kegg
    │       │   ├── ncbi_nr
    │       │   └── vfdb
    │       ├── mmseqs2_taxonomy
    │       │   └── gtdb
    │       ├── prokka
    │       │   └── logs
    │       └── rgi
    │           └── logs
    ├── assembly
    │   └── 1.2.1
    │       ├── megahit
    │       └── predictFlavor
    ├── binning
    │   └── 0.5.0
    │       ├── contigCoverage
    │       ├── contigMapping
    │       ├── genomeCoverage
    │       │   └── logs
    │       ├── metabat
    │       └── readMappingQuality
    ├── config
    │   └── 0.1.0
    │       └── parameters
    ├── fragmentRecruitment
    │   └── 0.1.0
    │       └── mashScreen
    ├── magAttributes
    │   └── 1.0.1
    │       ├── checkm
    │       │   └── logs
    │       └── gtdb
    │           └── logs
    ├── metabolomics
    │   └── 0.1.0
    │       ├── carveme
    │       │   └── logs
    │       ├── gsmmJson
    │       │   └── logs
    │       ├── gsmmTsv
    │       │   └── logs
    │       └── memote
    │           └── logs
    ├── plasmids
    │   └── 1.0.0
    │       ├── MobTyper
    │       │   └── logs
    │       ├── PLSDB
    │       │   └── logs
    │       ├── PlasClass
    │       │   └── logs
    │       ├── SCAPP
    │       │   ├── coverage
    │       │   ├── logs
    │       │   └── readMapping
    │       ├── ViralVerifyPlasmid
    │       │   └── logs
    │       └── filtered
    └── qc
        └── 0.3.0
            ├── fastp
            ├── jellyfish
            └── nonpareil
```

Supplementary Figure 4: Example directory output structure of one Toolkit sample run with the aggregation step.

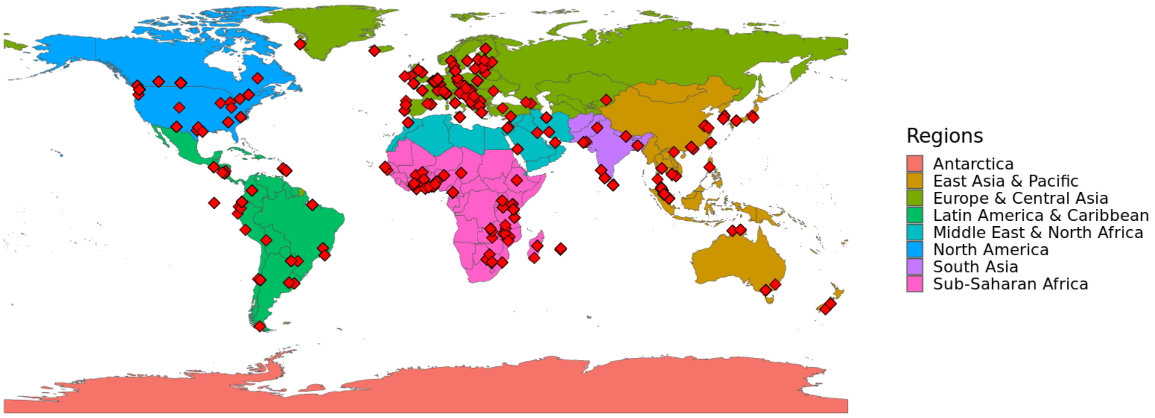

Supplementary Figure 5: World map colored according to the World Bank.

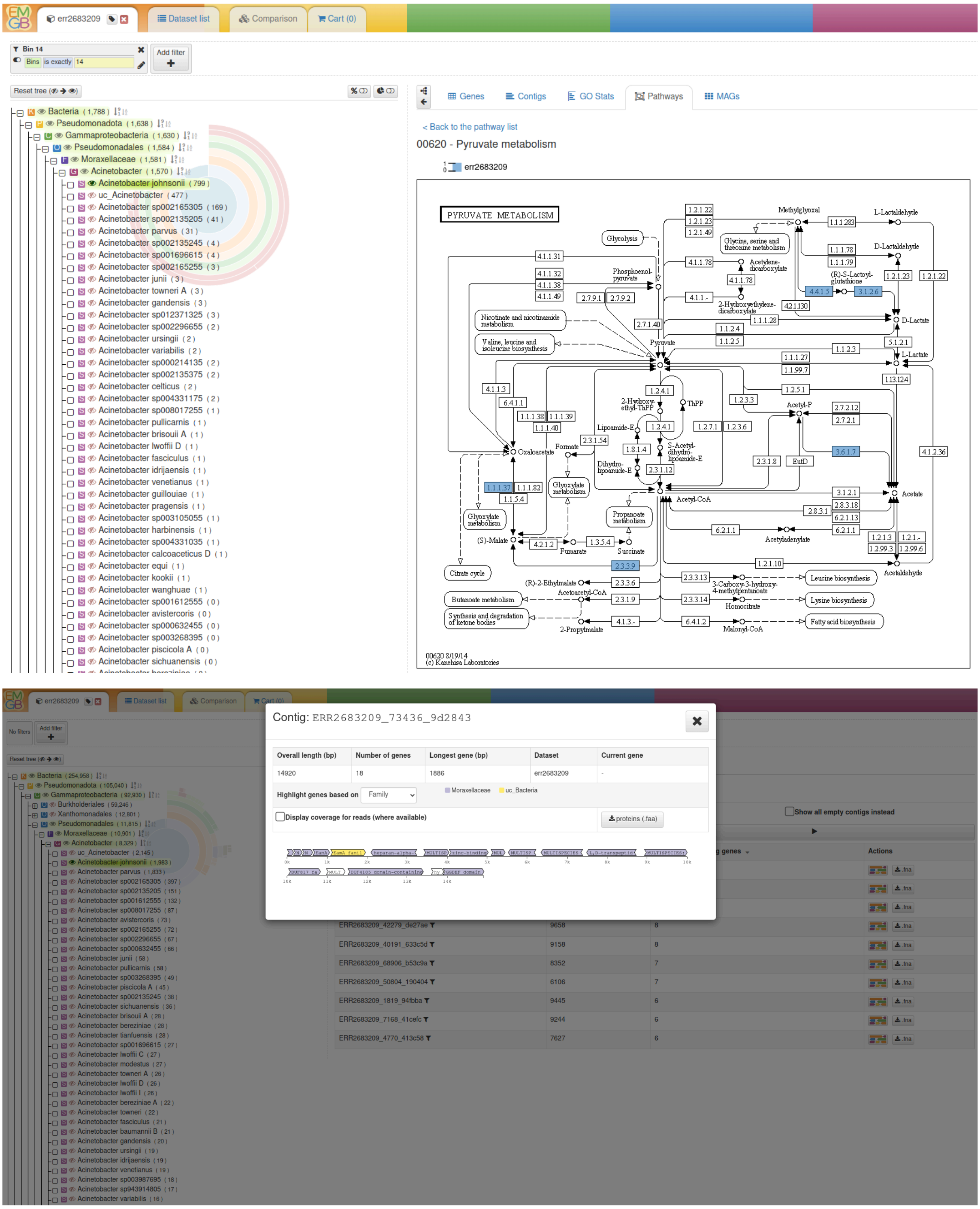

Supplementary Figure 6: Screenshots of the Exploratory MetaGenome Browser (EMGB). The upper screenshot displays a filtered set of genes and their occurrence in the Pyruvate Metabolism. The screenshot at the bottom shows genes that can be inspected by the contig viewer.

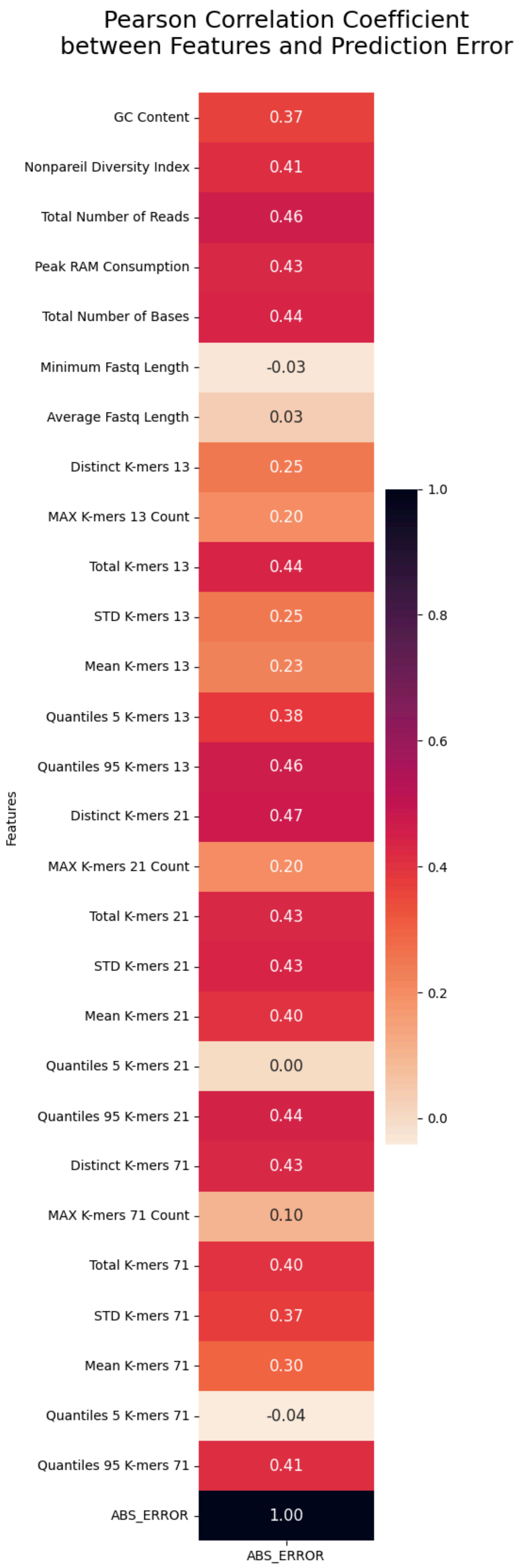
Pearson Correlation Coefficient
between Features and Prediction Error
Features
GC Content 0.37
Nonpareil Diversity Index 0.41
Total Number of Reads 0.46
Peak RAM Consumption 0.43
Total Number of Bases 0.44
Minimum Fastq Length -0.03
Average Fastq Length 0.03
Distinct K-mers 13 0.25
MAX K-mers 13 Count 0.20
Total K-mers 13 0.44
STD K-mers 13 0.25
Mean K-mers 13 0.23
Quantiles 5 K-mers 13 0.38
Quantiles 95 K-mers 13 0.46
Distinct K-mers 21 0.47
MAX K-mers 21 Count 0.20
Total K-mers 21 0.43
STD K-mers 21 0.43
Mean K-mers 21 0.40
Quantiles 5 K-mers 21 0.00
Quantiles 95 K-mers 21 0.44
Distinct K-mers 71 0.43
MAX K-mers 71 Count 0.10
Total K-mers 71 0.40
STD K-mers 71 0.37
Mean K-mers 71 0.30
Quantiles 5 K-mers 71 -0.04
Quantiles 95 K-mers 71 0.41
ABS_ERROR 1.00
ABS_ERROR
1.0
0.8
0.6
0.4
0.2
0.0

Supplementary Figure 7: Pearson correlation coefficient between features and prediction error.
